# Identity Construction and Community Building Practices Through Food: A Case Study

**DOI:** 10.3390/bs15121675

**Published:** 2025-12-03

**Authors:** Martina Arcadu, Elena Tubertini, María Isabel Reyes Espejo, Laura Migliorini

**Affiliations:** 1 Department of Educational Science (DISFOR), University of Genova, 16121 Genova, Italy; 2 Department of Psychology, Pontifical Catholic University of Valparaíso (PUCV), El Bosque 1290, Viña del Mar 2431200, Chile; 3 Department of Developmental Psychology and Socialization (DPSS), University of Padova, 35131 Padova, Italy; elena.tubertini@phd.unipd.it

**Keywords:** food practices, migrant communities, identity construction, empowerment, qualitative case study, community psychology

## Abstract

The present study explores the role of food as a symbolic, material, and relational device in identity construction and community processes. This study draws on a qualitative case study of a community-based social restaurant located in a mid-sized city in central-northern Italy. The initiative’s objective is to promote the social and labor inclusion of migrant women through training and experiential programs. The research, conducted over a period of nine months from October 2024 to June 2025, was based on a participatory qualitative design, which integrated semi-structured interviews, ecological maps, photointervention, world café, and affective cartography, involving 35 participants including operators, trainees, local community members, and politicians. The results demonstrate the multifaceted role of food practices at the restaurant, which serve to strengthen internal relationships, regulate community life, construct intercultural narratives, and establish spaces of recognition and agency for the women involved. Moreover, the restaurant has been shown to have the capacity to influence the broader social representations of migration in the urban context, thereby promoting processes of cohesion and belonging. It is evident that food-related activities manifest as quotidian micro-political practices, which have the capacity to subvert stereotypes, recognize frequently unseen abilities, and generate new forms of inclusive citizenship. The present study underscores the transformative capacity of initiatives that employ food practices as innovative instruments for fostering empowerment; well-being; and social participation; through the third element of food. The limitations and future prospects of the present situation are discussed; with particular reference to the need to ensure continuity and institutional sustainability for similar experiences.

## 1. Introduction

Within the domain of migration, food assumes the role of a potent medium through which individuals and groups engage in the negotiation of meanings, roles, and a sense of belonging (Gerber & Folta, 2022). In this perspective, it can be argued that, within the migratory context, food can be understood not only as a means of preserving cultural identity (Arcadu et al., 2025b), but also as a common project capable of generating meaningful and transformative connections with both the host community and the community of origin. At the individual level food traditions, practices, and representations constitute a fundamental core of identity of migrants and this offers privileged access to understanding their symbolic and relational worlds (Parasecoli, 2014; Simon-Roberts, 2025).

Food practices operate as a material field in which complex identities are reflected and co-constructed (Chapman & Beagan, 2013; Avakian & Haber, 2005; Miranda-Nieto & Boccagni, 2020). In this regard, food has been shown to negotiate belonging and boundaries between groups (Montero & Gross, 2023; Ren & Fusté-Forné, 2024; Arcadu et al., 2025a; Arcadu & Migliorini, 2024). The consumption of traditional food can be regarded as a symbolic act of resistance or a means of anchoring identity in the host country for migrants. In this sense, the literature refers to the construct of food acculturation as the process through which individuals or groups acquire and modify their food practices, preferences, habits, and cultural meanings associated with food in response to social interactions and cultural contacts they encounter in the new context (Satia-Abouta et al., 2002).

Food also plays a crucial role on a broader level in observing social interactions at the community level (Calvo, 1982; Crenn et al., 2010; Fischler, 1988; Mescoli, 2014). It is important to note that food places, which include restaurants, urban gardens, and shops, are not merely spaces for consumption. Rather, they are actual “socio-material environments permeated by different ways of constructing the collective identities of migrants” (Miranda-Nieto & Boccagni, 2020, p. 1023). The extant literature highlights the capacity of such collective devices to promote empowerment, belonging, and agency in social contexts characterized by multi-ethnic coexistence (Beauregard et al., 2019; Alfadhli & Drury, 2018; Ballentyne et al., 2021).

Participation in community food sites, such as urban gardens, is considered beneficial for social, mental, and physical well-being (Kingsley & Townsend, 2006; Parr, 2007; Hawkins et al., 2013; Pitt, 2014), contributing to the adaptation process of migrants and supporting the process of belonging to a place and community (Sampson & Gifford, 2010; Tidball & Krasny, 2013; Gómez et al., 2015). This, in turn, has the effect of strengthening well-being and a coherent sense of self in transformative contexts (Ballentyne et al., 2021; Jetten et al., 2010; Iyer et al., 2009). Furthermore, food plays a pivotal role in facilitating interactions between migrants and local communities, thereby fostering active participation, recognition, and social cohesion (Lugosi et al., 2023; Webster & Forsberg, 2020; Rabikowska, 2010). Furthermore, community food spaces differ from commercial or profit-oriented premises in that they are conceived as relational environments in which social support, collective participation, and shared meaning-making can emerge (Veron, 2024). This distinction is fundamental to our study, as it is precisely the community-oriented nature of these spaces that makes the processes examined here possible.

Despite the growing attention to the role of food in migration processes and the community practices that derive from it, however, the literature has so far paid little attention to such places as transformative devices capable of fostering belonging, recognition, and empowerment among migrants (Simon-Roberts, 2025). There is a paucity of in-depth reflection on how these spaces, intended here as socio-material environments in which everyday food practices acquire identity-related meanings, are experienced, narrated, and co-constructed by the people who inhabit them, and on the relational and symbolic dynamics that underpin the everyday practices surrounding food (Clarebout & Mescoli, 2023; Pitt, 2014).

The present study aims to address these gaps by exploring the role of food and related practices as symbolic, material, and relational elements capable of activating community processes within urban food places. Beyond addressing a gap in the extant literature, this study follows the invitation offered by Alvesson and Sandberg (2013) and Okimoto (2014) to construct research questions that challenge taken-for-granted assumptions within a field. Rather than treating community food spaces as neutral settings, we problematize this implicit assumption and approach them as socio-material and relational devices through which migrants actively negotiate identity, belonging, and recognition. Adopting this perspective enables the study to illuminate how quotidian food practices generate forms of community participation and empowerment that are often overlooked in mainstream accounts of migration and integration.

To operationalize this objective, the study was guided by three research questions. First, we asked what role community food spaces play in the lives of migrants and how food-related practices contribute to shaping a sense of belonging and community building within the social restaurant. Second, we explored how participants negotiate their personal and collective identities through their everyday engagement with food in this context. Finally, we examined the co-construction of shared meanings about food-related places and their role within the wider local community.

The present article draws on a qualitative case study carried out in a social restaurant located in a medium-sized city in Northern Italy. We adopt a multilevel perspective that connects participants everyday food practices (micro level) with the relational and organizational dynamics of the social restaurant (meso level) as well as with the broader processes of identity construction, belonging, and community participation (social level). This framework allows us to examine how situated food-related actions become embedded into community and contribute to wider patterns of recognition, empowerment, and intercultural coexistence. By articulating these interconnected levels, the study positions food as a socio-material device through which community relationships and identity trajectories are shaped. Furthermore, the present study employs a qualitative and community-based participatory research (CBPR) approach, fostering collaborative and empowering relationships between community members and researchers (Bond et al., 2017; Lincoln, 1998; Israel et al., 1998; Langhout, 2003; Sánchez et al., 2021). The CBPR approach informed the co-design of activities, the iterative shaping of data collection moments, and the shared interpretation of emerging meanings with staff members and participants.

## 2. Materials & Methods

### 2.1. Context

This case study was conducted in a social restaurant, established through the initiative of a local non-profit organization promoting the integration of migrant women. Social restaurants, also known as community or solidarity restaurants, are hybrid spaces that operate at the intersection of catering, social inclusion, and community development. They typically combine the provision of meals with broader objectives such as vocational training, social reintegration, and the empowerment of marginalized people, including migrants, women, and people in socioeconomically vulnerable situations (Sousa et al., 2021). The development of social restaurants in Europe is linked to the expansion of the social and solidarity economy during the 1990s and 2000s, particularly within the growth of social cooperatives and Work Integration Social Enterprises (Defourny & Nyssens, 2010). These contexts often operate within the third sector or cooperative structures and seek to create inclusive environments where food becomes both a material resource and a relational tool. In line with the principles of community psychology, they serve as enabling contexts where individuals are offered opportunities for participation, capacity building, and recognition. The primary objective of the initiative is to facilitate the social and professional integration of migrant women in vulnerable socio-economic circumstances through integrated training, employment, and active involvement in community life. The program is designed to span a period of four months and is grounded in an experiential learning model. The restaurant is a hybrid space that combines catering and community practices, as well as a place for intercultural encounters open to the public. It organizes public events, workshops, cultural initiatives, and participatory activities.

### 2.2. Study Design

The present study employs an interpretive qualitative design (Stake, 1995) and our broad conceptual orientation is informed by Mead et al.’s (1966) epistemological view that social reality is constructed through interactions and meaning-making processes. The objective is to generate a theoretically informed understanding through an in-depth analysis of the case under examination. The case study is justified by the need to understand a complex phenomenon in its real context (Yin, 2014). Social restaurants articulate social, economic, logistical, and community dimensions that require in-depth and contextualized analysis. The case was selected for its representation of a situated social configuration of food practices, community relations, and empowerment pathways in a migratory context. Indeed, experiences of food establishments run by and with migrants have been shown to play a key role in promoting processes of community resilience (Arcadu & Migliorini, 2024) and collective identity negotiation (Ballentyne et al., 2021; Alfadhli & Drury, 2018).

The analytical focus was on the dynamic set of practices, interactions, meanings, and narratives that traverse the restaurant. The research design incorporated a variety of data collection techniques in accordance with a methodological triangulation logic, with the objective of capturing the plurality of voices and enhancing the ecological validity of the study. The selection of these techniques was made with a view to their alignment with the participatory and co-constructed approach that guided the entire process. The detailed description of the narratives and themes that have emerged shows that the results can be transferred to work contexts similar to those in this study (Stake, 1995), i.e., those that share social, cultural, and territorial conditions comparable to those of the participants, as well as characteristics specific to their migratory trajectories. The possibility that these results may be useful for other experiences lies more in contextual similarity (where food and community practices revolve around migration and the solidarity economy) than in populations with different institutional dynamics. The design process was characterized by a flexible and iterative approach, guided by the emergent rhythms and relationships that manifested within the field. This methodology aligns with a conception of research as an ongoing activity that is firmly embedded within its contextual milieu (Welch et al., 2019).

### 2.3. Data Collection and Procedure

The preparatory phase of the study began in October 2024 and involved establishing contacts with the restaurant, co-planning the activities, and organising the logistical aspects of the project. The data collection process commenced only after receiving ethical approval from the Ethics Committee for Research in February 2025 and took place thereafter until June 2025.

Participants were recruited through a combination of purposive and snowball sampling, with the active collaboration of staff. Given that the study involved a pre-existing natural group, theoretical saturation was not applied in a strict sense; instead, we focused on deepening the situated understanding of the group’s shared experiences. The first author was responsible for data collection, benefiting from extended access to the field and a relationship of trust that had been established over time with the participants.

Five different techniques were used:

Semi-structured individual interviews were conducted with eight staff members out of a total of ten operators active at the time of the research. All staff members active at the time of the research were invited to participate; however, two were unable to take part due to temporary unavailability related to personal and organisational circumstances (e.g., maternity leave or job changes). The final sample of eight participants therefore represents the large majority of the staff and includes all the key professional roles, ensuring adequate coverage of the different perspectives relevant to the study. The interviews, which lasted an average of one hour, were conducted in Italian and followed a flexible outline divided into five thematic areas related to the community context in question: (1) the meaning of food; (2) relational and community dynamics; (3) the role of the restaurant in the local context; (4) personal and collective changes; (5) connections between food, migration, and inclusion. The interview underwent iterative refinement limited to the wording of individual questions, which were simplified or reworded if they proved difficult to understand, without however changing the overall thematic structure.

Ecological maps were proposed to three key staff members to visualize the ecological dimension of the project and the relationships with other local actors in a participatory manner (Migliorini et al., 2025; Pfefferbaum et al., 2013). The choice to involve only three professionals was intentional: they were the only actors with a comprehensive overview of the restaurant’s organisational functioning and its relational connections with the wider community. The activity involved the construction of a graphic map representing the density and quality of restaurants’ connections with local institutions, associations, and informal networks. The maps were analyzed using a qualitative approach, focusing on the identification of key actors, the density and directionality of relational ties, and the perceived relevance of each connection. The results were then placed in dialogue with the themes that emerged from other tools used, allowing for a triangulated understanding of the relational dynamics surrounding the restaurant.

Photointervention is a participatory visual technique developed in the field of social and community psychology (Cantera, 2010; Alencar-Rodrigues, 2016), aimed at promoting critical awareness, collective reflection, and transformative processes on social contexts and dynamics. Two photointerventions were organized with two distinct groups of women participating in the program in two separate stages: one with the trainees active at that time (t1) and one with former trainees (t2). Each session lasted approximately 4 h. Participants were asked to take photographs that answered the question: According to your role in the restaurant, what meaning does/did food have? At the end of each session, the images were shared and discussed collectively, and the participants also created a final collective product, reflecting together, through a generative question, on how the material they had produced could be used and re-elaborated.

World Café and Affective Cartography involved a diverse group of migrant women, institutional representatives, volunteers, and local citizens. The World Café provided an initial opportunity for collective discussion in a horizontal and informal setting (Löhr et al., 2020). The discussion tables led by facilitators were organized around three central themes: (1) defining the characteristics of a “nurturing place” in an urban context; (2) reflecting on spaces that promote the reception and inclusion of migrants; (3) reflecting on the relationship between gender, food, and social ties in community contexts. The results that emerged from the discussion tables were collectively shared and elaborated in plenary, after which the entire group co-constructed a shared affective map representing all the nourishing places mentioned and visually mapped them onto the city.

All activities were audio-recorded with the participants’ permission and fully transcribed. Throughout the data collection process, the first two authors kept a shared reflective journal and systematic field notes, which were used to document emerging dynamics in the activities, monitor our positioning and reflections developed in dialogue with the participants, and co-constructed decisions in subsequent phases.

For the Photo Intervention, the photographs were collected, digitized, and systematically catalogued together with the corresponding group discussions. Similarly, the World Café conversations were audio-recorded, transcribed, and linked to the notes produced on each discussion table. The maps were photographed, digitized, and accompanied by detailed field notes documenting the meaning-making processes that occurred during their construction. It should be noted that, as this was an intercultural and interdisciplinary research team, data were analyzed collaboratively, and the interpretations were contrasted and legitimized during the process of returning information to the group of participants.

In all techniques, the study followed a CBPR-oriented approach that involved participants in a meaningful way, including sharing discussion topics, collectively interpreting emerging meanings, and co-designing subsequent activities. This ongoing dialogic process also generated the idea of creating a public exhibition for a local festival on migration. This initiative emerged from the participants’ own interest in giving visibility to their experiences, illustrating how the research progressively became a space for collective reflection and community-oriented action.

### 2.4. Participants

The research design involved a total of 35 participants, who were divided into subgroups according to the different techniques used. Table 1 presents the sociodemographic characteristics of the staff members who participated in the interviews.

As illustrated in Table 2, the first group comprised trainees currently enrolled in the training program (t1), while the second group consisted of former trainees who had previously completed the program (t2). The division facilitated the investigation of the transformations wrought by the case in question.

Seventeen people took part in the World Café meeting and affective mapping workshop. Their socio-demographic characteristics are presented in Table 3.

### 2.5. Positionality

The primary author is an Italian community psychologist with training in qualitative research applied to migration contexts. She entered the field without any previous collaborations, which required a significant investment in building relationships of trust. The researcher’s position was consistently included as an epistemologically relevant element in data collection and analysis. Her awareness of her own socio-cultural identity, including markers of belonging to privileged groups in terms of ethnicity, citizenship, gender, educational background, and socio-economic status, guided a reflective stance aimed at valuing the voices of those involved and critically questioning the conditions in which those voices take shape.

### 2.6. Data Analysis

The analysis of the data was conducted using thematic (Braun & Clarke, 2006, 2021) and narrative thematic analysis (Riessman, 2008). An interpretive approach was used and in this sense, thematic analysis procedures were integrated with a narrative reading, focusing on the ways in which participants construct meaning, agency, and positioning throughout their stories and visual productions. The integration of thematic and narrative approaches enabled a shift from the empirical categories to more latent interpretations oriented toward the symbolic, affective, and micropolitical dimensions of food practices. This integrated approach facilitated the interpretation of practices described as forms of care, resistance, or identity renegotiation in the context of migration: for example, the reference to the preparation of specific dishes was interpreted as a way of caring for oneself and others, of reaffirming one’s value in a work context, and of renegotiating one’s identity as a migrant woman within a new social environment.

The analytical process was conducted through a series of iterative comparisons between the first two authors, who independently coded the materials and subsequently met to discuss coding discrepancies, compare interpretations, and refine the emerging thematic structure. Through this consensus-building process, the authors jointly validated the final themes, and any remaining uncertainties were resolved in consultation with the other researchers involved. To facilitate the organization of the materials, NVivo 15 software (QSR International, Melbourne, Australia) (Allsop et al., 2022; QSR International, 2014) was used collaboratively. The analyses of the materials were organised according to the following stages: an initial phase of familiarisation with the data carried out independently by each researcher, followed by the systematic generation of initial codes produced separately by the two coders. These codes were subsequently grouped into preliminary themes, which were then jointly discussed to identify shared thematic areas. The final thematic areas were defined and named through further refinement of the themes that had emerged.

Although the preliminary analytic work was conducted by the research team, the data analysis remained aligned with the CBPR framework through ongoing cycles of dialogue and co-interpretation with participants. Preliminary results were returned to participants in a dedicated feedback session, where emerging themes and interpretations were discussed collectively. This moment of dialogical validation allowed participants to refine, challenge, expand the initial analytic insights, and also generated new meanings and directions for the ongoing project.

### 2.7. Ethical Considerations

The present research project has been approved by the Ethics Committee for Research of the University of Genoa (CERA, N. 2025/35). Prior to participation, all subjects were informed about the objectives of the study, the methods of participation, and the use of the data collected. Informed written consent was obtained from all subjects before they took part in the research. The data was managed according to a protection plan that involved storing it in password-protected folders, using codes to replace any personal references, and removing any potentially identifying details in order to minimize the risk of re-identification.

## 3. Results

### 3.1. Interviews and Ecological Maps by Professionals

The first section of the results presents the conclusions drawn from the interviews conducted and the ecological maps developed with internal staff. The preliminary analytical insights, initially identified by the research team, were subsequently shared with participants and further refined through collective discussion.To guide the reader, we first describe the four thematic areas that emerged from the analysis of the interviews, which illustrate the role of food practices in promoting internal relationships, acting as operational and strategic tools, opening channels of interaction with the community and the territory, and contributing to the transformation of broader social representations, generating processes of recognition and empowerment. A summary of the main themes is presented in Table 4.

We then present the ecological maps, created with staff members, which complement and expand on these themes by visually representing the organizational, relational, and territorial dynamics that shape the functioning of the social restaurant. A summary of the main themes is presented in Table 5.

#### 3.1.1. Food as a Central Feature of the Project: From Personal Experience to Social Interaction

The interviews reveal that food constitutes the symbolic and material center around which the entire identity of the project takes shape. The staff describe food as a vital aspect of community life, a space that fosters cohesion, shares narratives, and reinforces shared values. Food and food-related practices are described primarily as a language that connects different people and facilitates communication even in the absence of a common verbal code. As one of the workers observes: “Food is a relationship, a form of communication. We have found a way to communicate. Because there are so many different countries, and we don’t know each other’s languages. Instead, we talk without speaking the same language, but with food. Each woman has her own dish and talks about herself, about her identity (Int3)”. Or food is described as a language with customers who come from outside, as the dining room assistant explains: “When I talk about the Moroccan dish, I’m not just talking about myself, I’m talking about the Moroccan people and their culture. At the restaurant, I make sure that customers remember that it is not just about eating good, enjoyable food, but that it’s also something else entirely: food gives soul, it heals the soul. (Int4)”.

Food is deeply intertwined with the daily life of the project, strengthening the sense of belonging, as the coordinator points out: “Food is really what unites us all, at all times. Even I, who work in the office, arrive, taste something, and there is always the smell of something coming from the kitchen, because we work in the dining room right next to the kitchen. (Int1)” In addition to its informal dimension, food assumes a ritualized and formalized role in the training program. It is interesting to note that self-presentation through the creation of a meaningful dish constitutes one of the most intense moments of the entire program, as it explicitly links who participants are with what they produce, making food a concrete vehicle of identity expression, as the chef trainer explains: “Food is a bit like history. When we ask new trainees to introduce themselves, they come to this day called ‘Who am I?’ and introduce themselves by preparing a dish: this is a way of getting to know people, not the dish itself or the technique, but what it represents for that person. (Int8)” Furthermore, sharing food experiences can open spaces for dialogue on intimate or complex issues, as one interviewee explains: “Food is also a way to get to know each other better, to chat through food, and that has led us to talk about topics we wouldn’t have touched on if it hadn’t been for what we were cooking (…) During the pasta lesson, an intern was telling me about her husband, then we talked about my husband too; the other intern heard and joined in the conversation. So, yes, we got to know each other better starting from the pasta we were making, which led us to talk about cooking at home; something perhaps very simple, which may seem trivial, but which we might not have had the opportunity to talk about otherwise. (Int3)” The initial section underscores the way food assumes the roles of both an identity and a relational instrument within the project, thereby facilitating the establishment of interpersonal connections and fostering openness towards others.

#### 3.1.2. Food as an Operational and Regulative Tool Within the Internal Community of the Project

In this second dimension, food and related practices are described as having an operational function within the project, through which group dynamics are regulated, critical issues are addressed, and strategies for coexistence are built, shaping the daily life of community work. In this sense, food acts as a third device: a material and symbolic tool that invites individuals to engage with each other, make decisions, manage differences, and define common ways of coexisting and collaborating. In the context of community work, a “third device” refers to an artefact, practice, or shared reference point that enables participants to engage with one another, regulate group dynamics, and co-construct common frameworks for collaboration. The first area in which this function manifests itself is the organization of work in the kitchen. Around daily food practices, shared rules are constructed, team-building strategies are tested, intercultural conflicts are addressed, and educational spaces are generated. The shared goal is to build a team: “Three people here and two people on the other side is not a team, it’s not what we want in the kitchen. You have to build a group. We work a lot with team building, with shared rules in the kitchen, with technical classes, with the chef, always together. (Int6)”.

Building a collaborative environment also involves addressing the challenges posed by cultural diversity, which can lead to misunderstandings in the kitchen: “Those who don’t know the language very well find it difficult to express themselves, and this makes things very difficult. (Int5).” In addition to interpersonal tensions, the challenges of shared work for the staff become visible and therefore addressable: “It helps us to be clear about our goal with respect to the program: we don’t have to make this space harmonious at all costs. We don’t have to become friends, but I have to point out that when you go to work, if you act this way, it comes back to you. The kitchen is a laboratory. (Int8)” This reflection represents a particularly powerful insight, as it emphasizes the importance of maintaining the collective goal of the project as a third general reference point, which goes beyond personal affinities or individual tensions. Thanks to reflections on food practices, it is thus possible to shift the focus from interpersonal dynamics to the shared goal of supporting the restaurant as a socially oriented space. Staff can thus articulate a process of constant accountability that helps regulate daily interactions and align different contributions toward a common mission.

The second theme that emerged relates to how food and food practices become operational strategies, formalized and continuously practiced by the team. A first strategy consists of creating informal moments of food sharing—such as lunches or free access to drinks and food—which reinforce the sense of welcome and community and open relational spaces even outside structured activities: “There is a benefit: free coffee, free water. (…) Lunch time, for example, after class, opens a moment of sharing that is also very important for those who did not participate in the class, where people from outside can also come. (Int2)” A second approach is the strategic use of the playful aspect of food. As the trainer chef explains, the humor and lightheartedness associated with cooking become tools for maintaining motivation: “My sous-chef and I create a playful atmosphere in the kitchen, because it’s better than silence… I mean, joking around helps a lot! You have to keep the mood up, because (the intern) is someone who woke up at 6 a.m., took four children to different schools, picked them up and then came here. (Int8)” A third strategic use of culinary practices is to use them as a means of dialogue and building shared knowledge: “(…) in the kitchen, while we’re doing the activity together, there’s a lot of dialogue, and even those who didn’t know how to cook churros, for example, now know how to make them, so the strength of the project is precisely the sharing, the exchange of knowledge and enriching each other and producing something new through collaboration: this is what you notice most in the kitchen. (Int4)” Finally, another strategic use is culinary storytelling as an intentional and collective practice. At the restaurant, every dish is an opportunity to give voice to individual and collective stories, to value the cultures of the participants’ origins and to make the training process visible, thereby turning food into a medium through which identities are expressed, negotiated, and shared. As one participant explains: “We have several meetings with the trainees and the head chef, where she gets inspiration to create this menu based on the trainees’ backgrounds; and this storytelling of cultures (through the dishes) is so central to the daily work that the whole team is involved in researching and creating these narratives. (Int1)” Every three months, the narratives of the dishes are constructed and refined during service: “there is this task: this is the menu, now build the narrative around it.’ (All this) reinforces these processes and each person becomes a bit of an ambassador for the narrative itself. (Int7)” Narratives also contributes to developing a decolonial approach, shifting the focus from technical performance to human experience and the valorization of the subjectivity of those who live in that community. In a context such as Italy, where food takes on a strong identity and normative value, the project works to deconstruct stereotypes and cultural hierarchies and “manages to create this dialogue starting from the story of the person; I don’t come to this restaurant because I want to taste the best Pakistani dish, but to support this woman who is committed to integrating herself and finding work. This is what the restaurant manages to do (Int4)”. The incorporation of food as a narrative strategy thus becomes a pivotal element, facilitating the convergence of values, educational, and community dimensions of the work experience through continuous operational reflection.

#### 3.1.3. From Kitchen to City: Exploring and Transforming the Larger Community Through Food

A further level of analysis emerged from the interviews concerning the connection between the restaurant and the local area, both in terms of its integration into the urban fabric and its transformative function regarding the relationship between migration, citizenship, and community.

In this case, food functions as a catalyst for openness, exploration, and change. From an individual standpoint, employment at the restaurant has furnished the operators with novel insights into the urban milieu: “Here, we talk about lots of places; I discovered a restaurant, for example, which serves Chinese food, which is a bit unusual. Now I have a new map in my head of places that are interesting to try and discover. Much more than I had in my previous jobs. (Int2)” Working at the restaurant becomes an opportunity to rethink urban geography and forge new connections. One volunteer refers: “I was asked to go and buy something, I went to buy bread at the bakery, I’ve passed by a thousand times, I’d never noticed it. This has helped me a lot to be able to place things that I couldn’t place before. (Int3)” The relationships built up through the restaurant’s routine and physical proximity to other people in the area are also important: “We have this relationship with the bar opposite, or with the woman who cleans the library, who came in to ask for information for her sister who was arriving from the Ivory Coast. (Int2)” Finally, food facilitates conversation with outside customers. As the waitress observes: “Maybe… someone tastes something and then they’re curious (and ask), ‘Can you repeat the name, I want to make it at home, what was in it?’ It’s a kind of test, which becomes central to the conversation. (Int4)”.

Secondly, the words of the staff highlight the transformative potential that the project has for the wider community. The customer experience at the restaurant is not limited to the purely gastronomic dimension, but triggers deeper transformative processes, as one waitress explains: “When [a customer] comes to eat at the restaurant, they don’t just come for the food, but to try something they’ve never tried before. It has happened many times (that a dish was not liked), but then customers come back because they understand that they didn’t just come to eat. (Int8)” The culinary experience it offers become concrete references that can influence the representation of migrants, giving them humanity and dignity. The program director says: “Before, there was nothing about migrants in the press, they were almost invisible. Now there is, because at the restaurant this voice is strong, very present, very solid. So now I think people have a physical reference point. It’s a very psychological thing, this restaurant gives a lot of humanity, dignity, curiosity, beauty, it’s celebratory, it’s not sad, it’s a very positive voice. And this was absolutely not there before. (Int7)” Dialogue with the community on important issues is kept alive thanks to daily activities, as the director reports: “There is also co-working, which allows for continuous dialogue between the restaurant and the entire community. I see that people are talking more, and this is an important change because addressing this issue at the table [the phenomenon of migration] cannot be avoided, it is real, we are here, there is a huge community that needs to integrate. Integration is precisely communication between the two sides, it’s not, ‘I don’t eat pasta,’ you have to try it first. So, it’s important because it is the beginning of something. (Int7)” The restaurant therefore takes on value as a social device, promoting encounters and the construction of new shared frames of meaning.

#### 3.1.4. Food as a Space for Agency: Practices of Self-Determination and Identity Transformation

The final theme emphasizes the transformative influence that culinary endeavors at the restaurant exert on a broader scale, functioning as a symbolic domain of agency and self-expression for the trainees.

The initial thematic cluster at this level pertains to the potential for individuals to reaffirm their sense of identity through the medium of food, thereby rediscovering the value of their own culture. The restaurant is a space that facilitates authentic expression: “We have a batter on the menu that in my culture is only made by high-class families. So, I’m proud because I celebrate birthdays with huge batters at the restaurant. I am proud of the food and tradition because before we just ate, we didn’t think about these things. Now with this restaurant, I think there is an origin and a story to tell. (Int4)” Finding oneself allows for a deeper change in one’s relationship with the social context: “I think that before I felt I had to become Italian in every way. I discovered while working here that this is completely a lie. I am very proud of the differences I bring to my work and to society. So yes, I have changed a lot… I have gone back to how I was before. I feel very much like myself, which is very nice. (Int7)”.

The second core principle emphasizes the empowerment of migrant women trainees participating in the project. The work undertaken at the restaurant has resulted in a shift in the perception of the kitchen as a space for invisible, privatized and unrecognized gender roles. Instead, it has become a setting for profound social recognition, even for those daily and ‘minor’ tasks that are reinterpreted as professional and cultural skills. This transformation in perception is indicative of a broader process of social change, whereby the social position of those involved is being redefined. The dining room manager employs an efficacious food metaphor to elucidate: “Before, when women told me about the food they cooked, it was worth nothing. But then they realized that the dishes they used to make back home, including everyday street foods, are actually great [valuable]! (Int5)” In addition, a former intern, now a restaurant manager, recounts how restaurant represented a space in which to give shape and direction to the practical knowledge she already possessed: “It gave me the right ideas. Because it came at a time when I was asking myself, ‘What am I doing here?’ and then I said to myself with this restaurant, ‘I can’t stop now.’ Here, I found a place to put the experience I had. (Int4)”.

Finally, the third thematic core highlights the power of cooking to enable the recognition of others as experts, subverting the hierarchies of knowledge typical of Western professionalization. The chef explains: “I follow the recipes, but if I am not sure, I ask how it’s done in your country: ‘Come and taste it, I want to know why you know better than me if the taste is the same!’. (Int6)” The intercultural cooking practices that are promoted and practiced at restaurant become deeply social and political symbolic devices. These devices have the power to promote new narratives about the value of differences and the transformative role of migrant culture in local society.

#### 3.1.5. Ecological Maps

**Figure 1:**
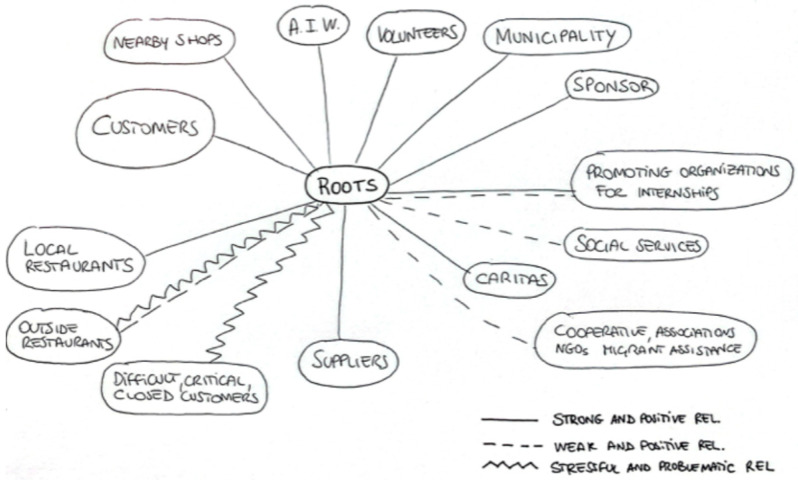
Joint ecological map of three key interviewees.

The ecological maps created after the interview by three key operators involved are presented together in Figure 1. The maps highlight some significant convergences in how the social restaurant is perceived as positioned within the broader community fabric. What emerges is the image of a fluid and interconnected device, capable of operating as an interface between heterogeneous social subjects. Among the actors mentioned are organizations promoting internships and job placement, the regional government, the municipality, volunteers, sponsors, suppliers, local restaurants, customers, social services, and local associations. Concurrently, there have been reports of critical or problematic relationships with specific commercial entities, local associations, or customers who are perceived as rigid or unwelcoming.

### 3.2. Photo-Intervention Experience with Migrant Trainees

The second part of the results focuses on the photo-intervention process conducted with active trainees (t1) and former trainees (t2). To guide the reader through the analytical process, we present the thematic patterns that emerged separately within each group, and then highlight the points of continuity and change that allow us to understand how the meanings attributed to food evolved during the training experience. The main themes are summarized in Table 6.

This section therefore complements the previous one by offering a visual and narrative overview of the personal and relational transformations associated with participation in the project. The thematic analysis revealed common themes between the two groups, both in terms of photo selection and final product. In addition, the analysis revealed variations in narrative style. These variations highlighted differences in terms of cohesion, experience, capacity for reflection, sense of community, and social responsibility.

One theme that emerged in both groups concerns the meaning of food as personal growth and future opportunities. Group t1 illustrated how shared food practices open new perspectives for the future. Personal growth, in the early stages of the training experience, takes on the meaning of humility, hard work, and patience “you have to know that we don’t know everything. We are discovering so many new things and humility is necessary and chef M. has a huge career, yet she comes here and talks to us as if we were friends. She teaches us with patience and humility (T1)” The narratives reveal that culinary learning requires a process of experimentation, error, and repetition, facilitated by teaching practices and a training environment based on respect and relationships in the kitchen. At the same time, personal growth is also articulated through happiness and a sense of self-efficacy derived from the awareness of having acquired new skills and achieved something independently. For group t2, the theme of personal growth also emerges clearly, albeit with a different nuance, linked to food practices such as transformation and rebirth.

**Figure 2:**
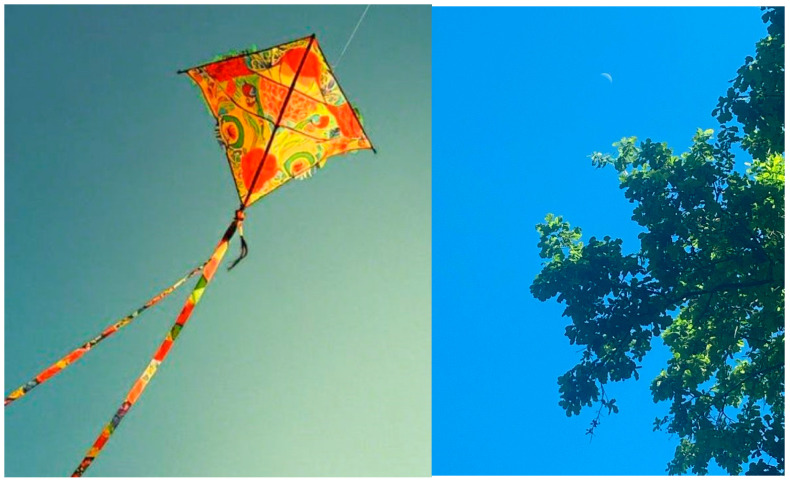
Food practices as transformation and rebirth (group t2).

Many of the women who have experienced migration, often characterized by feelings of loneliness, describe finding an opportunity for personal blossoming in their journey at the restaurant. In their narratives, food and food-related practices emerge as powerful agents of freedom to emerge from darkness, discover and rediscover oneself, as this former intern explains while showing her photos, visible in Figure 2: “This is me alone, scared and with so many dreams of flying, of moving forward, but in our culture it is not possible for a woman to leave the house, work or have dreams. I am a moon that [used to] always be afraid of the sun. But not now, now I have [come out] during the day, I am the moon in the daytime (T2)”.

In this perspective, cooking represents for the participants (t2) not only technical support and satisfaction in creating a dish, as emphasized by the group of trainees (t1), but also an empowering experience and a stimulus for independence and social inclusion (e.g., obtaining a driver’s license, finding a job, or learning Italian). This interpretation enriches the value of patience with that of stability (not only economic), allowing personal and professional goals to be achieved. One of the women explains, “My dream was to become a chef, but now it is important for me to be independent, for my children. I want them to live in freedom—which I didn’t have before, but now I have a little bit. This is also this restaurant for me, this bicycle and the flowers: we are women, different, beautiful, independent, and here the restaurant has given us all beautiful things. (T2)”. In particular, the photographs of the former trainees (t2) provide a more comprehensive and retrospective view of their journey, accompanied by deep gratitude for the project and the opportunities offered by the shared practices.

The theme of food as a relational and cohesive space emerges in the group of trainees (t1), who describe how cooking together with other women has created a dynamic that goes beyond the common benefit of productivity, but which nourishes a feeling of connection, building a community made up of bonds between peers. This concept is expressed in a more complex way in group t2, which, in showing their photographs and commenting on those of others, demonstrates a level of emotional maturity and group cohesion, as well as a keen awareness of the theme of food-related relationships. The theme can be summarized as flourishing with and for each other: “flourishing with” because, through food and cooking, a dynamic of contact and knowledge is established with the other woman, like a “second family” that can nurture, grow, and become a source of support. One recognizes oneself in the other, receiving support and protection from woman to woman. The friendships formed during the journey are described and represented in the photographs as “a gift I received, the hand, the heart, the wonderful companions saved me. (T2)” And then “blooming for,” because once stability is achieved, women have the skills and maturity necessary to offer roots to other people, both to one’s own children and family, and to the next project interns and the women with they share the educational journey, as this former intern shows: “We are all here, dry [branches]. The water we take and give to the restaurant to grow… and [thus] we give roots to our children. We [also] work for the restaurant, we sacrifice ourselves by being ambassadors… (T2)”.

The metaphorical utilization of food as a medium for self-rediscovery can be linked to the metaphorical transformation of a tree, which is not only stable and anchored to the ground, but also serves as a source of nourishment for others to cultivate their own goals. The practice of sharing food is therefore outlined as a driving force of love, which, through the sharing of the culinary preparation, strengthens the sense of family, emotional, and community belonging. Activities in the kitchen, which frequently necessitate all participants to attend, are regarded as a drive for cohesion, enabling them to overcome feelings of isolation and offering a foundation for emancipation. This process fosters their uniqueness, encompassing both cultural and social dimensions, and facilitates the liberation from predefined social roles when needed.

**Figure 3:**
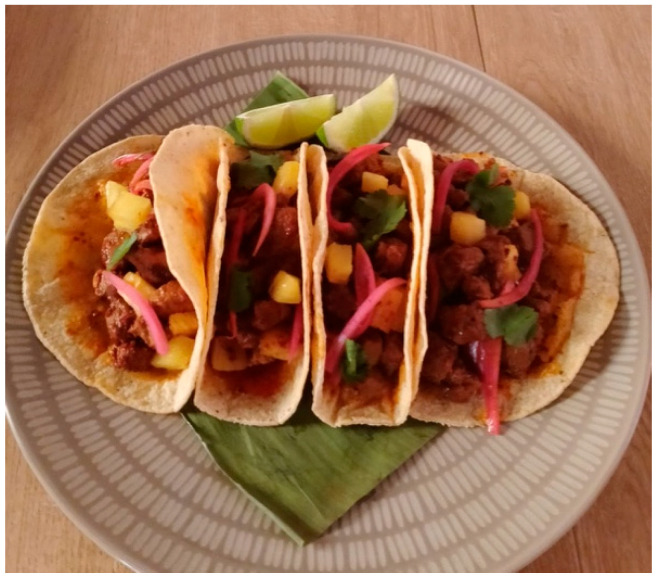
Food as harmony and richness in diversity (group t1).

This cultural aspect of food is best encapsulated by the theme of food as harmony and richness in differences, which emerged in the group of interns (t1). Around food, practices of sharing knowledge related to it are created, allowing everyone to express themselves in their diversity and, at the same time, to understand the richness of their differences. The cultural and knowledge exchange around food fosters a balance between the diverse backgrounds and experiences brought by the interns, as this woman explains while showing her photo (Figure 3): “This represents the flavors we have on the menu: different, very tasty, many, but they go well together. Diversity, even if it sometimes seems strange, the different colors, create an unexpected perfection. I’ve tried flavors that I had no idea could go together, I found them here. (T1)”.

**Figure 4:**
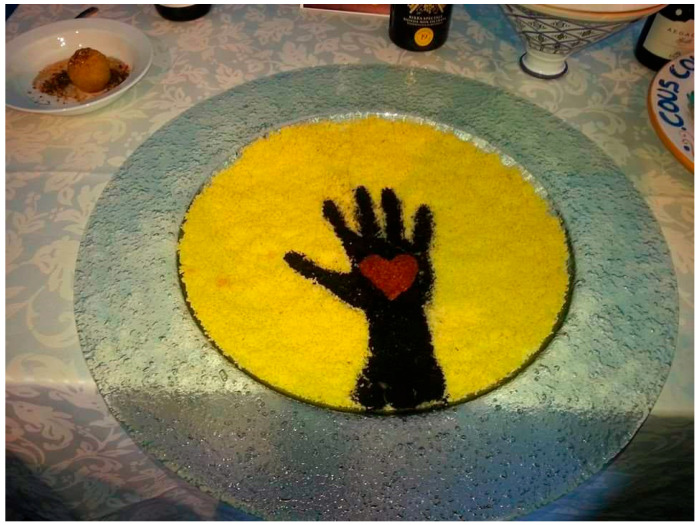
Food to nourish diversity and promote dialogue (group t2).

The opportunity to exchange ideas allows participants to understand that the priorities, meanings, and stories associated with food do not always match those of others. Food is the common thread running through all experiences, as is the identity of migrant women, which unites the participants not only in the present moment as a group, but also creates a continuity of identity over time, as new trainees join the program. The same theme is described in a similar way by group t2, which talks about food as a way of nourishing diversity and promoting dialogue. On the one hand, food emphasizes differences, promoting creativity and self-expression; on the other hand, it erases them, by placing everyone on the same level, overcoming cultural and personality barriers. As a former intern explains, showing a photo of a plate of couscous (see Figure 4): “Food is not for the mouth, I realized that it is us, among ourselves… you from one place, me from another, even if we don’t know how to speak, here [food] brings you together, even if you don’t know how to speak their language or understand their character in the kitchen, you are in another world, you find many friends, we taste, we do things. For me, cooking is a way that unites everything (T2)”.

The last thematic block concerns the relationship with the community and cultures, both with respect to the country of origin and the local Italian territory. As far as the interns (t1) are concerned, the importance of food in feeling at home and meeting others emerges. The restaurant, in fact, on the one hand “makes you feel at home through hospitality and food, (T1)” but it is also “an opportunity to share your traditions. (T1)” The practices and narratives shared around food and the dishes on the menu play a fundamental role in shaping this meaning of food as a bridge between cultures, as this intern clearly illustrates: “it’s a responsibility on the one hand, we have to communicate to an audience that is not used to eating these things that it’s a beautiful and important thing. I always keep in mind… here they told us, ‘We don’t make traditional food, but food that allows us to talk between two cultures through food,’ which is different. Because traditionally they wouldn’t be like that… there’s the flexibility to say, ‘My dish has to speak to the other person, for a first encounter between two cultures.’ That’s what this restaurant means to me. (T1)” According to former interns (t2), this theme is expressed in different ways, in food as representation, integration, and responsibility. One former intern describes the sense of responsibility to pass on to customers and the local community what she has learned: “In food, we transform who we are, our character, taste, love… everything goes into this dish that we create and give to the customer. (T2)” In conclusion, it emerges that shared food practices generate a combination of new flavors thanks to the addition of Italian ingredients, in a metaphor for social inclusion: “In Tunisia, we don’t have Parmigiano Reggiano because it’s typical of Italy. When I added the Parmigiano, I mixed things up and created something new right here at the restaurant… it became a new dish because it has a special taste. (T2)”.

**Figure 5:**
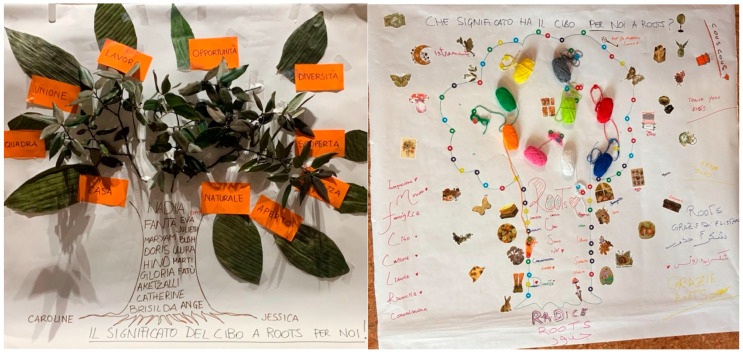
Final products of the two photo-intervention projects (group t1 on the left, t2 on the right).

The final discussion within each group brought out visual narratives, which led to a very similar final material product between the two groups of participants, namely two representations of trees. The group of trainees (t1) describes their tree, Figure 5, as unity and common roots in diversity: “We are all different, we have different dreams, we have different priorities, but there is always, for us women, there is always one thing that unites us. Like this tree, the leaves may be different, but the roots are always the same. (T1)” The tree of the former trainees (t2) shown in Figure 5 echoes many of the keywords and suggestions of the previous group, but adds a nuance related to independence, a sense of family, and freedom: “Freedom and trust, the trust that this restaurant has given us through food; through food there are so many other things, integration, family. (T2)” The convergence on the tree metaphor in both groups suggests a deeper symbolic pattern beyond the similarity of the object. In psychosocial terms, the tree is a powerful metaphor for identity that is both grounded and evolving. It evokes a connection to one’s origins (roots), the nourishment and relational support that enable growth (trunk and branches), and an openness to future possibilities (new leaves and directions). The recurrence of this image in two independent groups suggests that participants collectively interpret their experience in the project as a simultaneous process of rooting, expansion, and transformation. The tree becomes a shared, representative materiality articulating the dual movement of migrant identities, which are anchored in personal and cultural histories yet dynamically reshaped by interactions, practices, and opportunities encountered.

### 3.3. World Café and Participatory Affective Cartography

**Figure 6:**
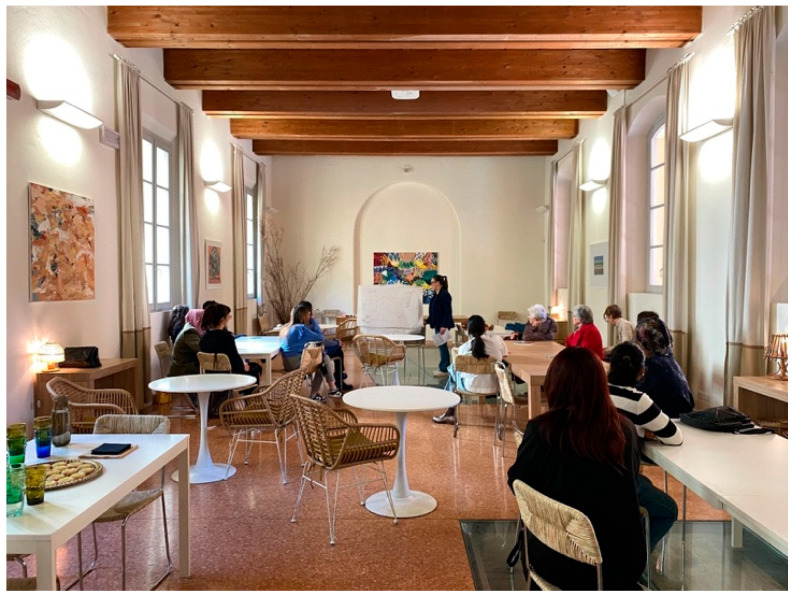
World Café and participatory affective mapping, on International Women’s Day.

The third and final section of the results focuses on the collective activities carried out during the World Café and the participatory affective mapping session, as illustrated in Figure 6. To ensure continuity with the previous analytical phases, this section explores how the themes identified at the individual and group levels translate into broader reflections on urban spaces, dining venues, and community cohesion. We first present the thematic insights that emerged from the discussion tables, then illustrate how these contributions were visually synthesized through the map. The main discussion themes that emerged are presented in Table 7.

The first discussion theme sought to define the characteristics of a place that nurtures in an urban context. A nurturing place was described as a space that supports, welcomes, and allows people to feel recognized, promoting self-construction and the formation of meaningful bonds. These reflections highlight that the concept of a welcoming place is associated with its relational, symbolic, and experiential potential. These are places where it is possible to “feel recognized” which refers to a deeper psychosocial process: the possibility of being seen as legitimate subjects within the urban fabric. However, obstacles were also identified that limit access to such spaces for the women participants: language barriers, relational insecurities, perceived mistrust on the part of local citizens, and a lack of knowledge of the urban area. These aspects underscore how access to urban spaces is also mediated by social representations and power relations. The restaurant was spontaneously cited as a concrete example of a public place that offers opportunities for exchange, thanks to its welcoming atmosphere and daily practices that value the culture of the participants.

The second theme focused on which spaces promote the reception and inclusion of migrants, with particular reference to the analyzed context. The discussion revealed a connection between urban accessibility, language barriers, and the actual possibility of feeling welcome. Large supermarkets are perceived as less demanding in terms of interaction and more linguistically safe: “I go to the supermarket because I don’t know the city. I don’t go out if I’m a migrant woman. And at the supermarket, I don’t have to talk to anyone.” These reflections highlight how the experience of inclusion in the city is mediated more by the social conditions that make these spaces navigable. The preference for supermarkets as linguistically safe contexts highlights how the fear of miscommunication shapes daily mobility, also producing subtle forms of self-isolation. The city market was then cited as one of the few places capable of fostering informal intercultural exchange. This also suggests that inclusion emerges where interaction is possible in everyday exchanges. Experiences of socialization linked to events promoted by associations or convivial moments within specific projects also emerged, including the restaurant, defined as “an exception.” However, these experiences are still fragmented, little known, and difficult to access except through informal networks. This fact points to the need for intentional, community-oriented infrastructure that actively reduces barriers to participation.

The third table worked on the theme of how food places can help build bonds in the community. The discussion highlighted differences between long-term residents and more recent migrant arrivals. Two types of places emerged: on the one hand, spaces evocative of family memories (such as pasta shops frequented by grandmothers or mothers); on the other, places run by women as examples of female representation in the food industry. For long-time residents, places linked to food and family memories evoke continuity and intergenerational belonging, reinforcing a stable sense of belonging to the place. For more recent migrants, however, food-related places run by women become reference points through which to imagine new possibilities. Institutional spaces, such as schools and parishes, were also mentioned, which promote socialization and intercultural openness through food.

Overall, the discussion provided a critical and proactive vision of the city, highlighting the need to rethink food places as meeting places, as social arenas where relationships are established, identities are negotiated, and forms of everyday citizenship take shape. Food venues emerge as micro-infrastructures of urban life capable of challenging isolation, redistributing opportunities for participation, and enabling encounters that might not otherwise take place. Rethinking these spaces as meeting points therefore means recognizing their potential to reshape the affective and relational geography of the city, transforming them into accessible, inclusive, and culturally significant environments where differences can coexist, be recognized, and create new possibilities for community building.

**Figure 7:**
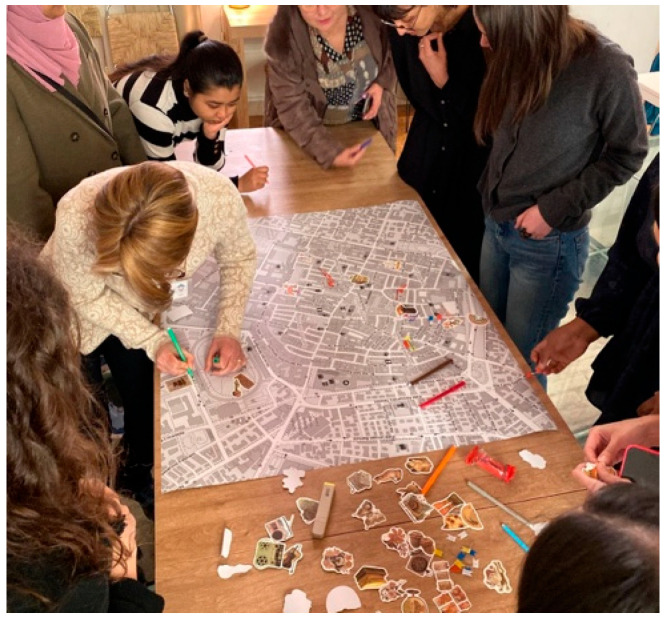
Collaborative construction of affective mapping of nourishing places in the city.

Based on the content that emerged from the three round tables, the group co-constructed an affective map of food places in the city, shown in Figure 7.

The map facilitated a visual summary of the experience and an exchange of localized knowledge, also allowing participants to discover previously unknown spaces. Significantly, the restaurant was represented with the word “rebirth,” signaling the transformative value attributed to the place and the experiences lived within it. Beyond its descriptive function, the map served as a psychosocial instrument that materialized the collective meaning attributed to the city by the group. Participants reconfigured the geography of the city by placing emotions, memories, and relational experiences in the urban landscape. They identified the places they frequent and those where they feel they belong. The affective map is a conceptual framework that captures and reinterprets the dynamics of urban space, emphasizing the active role of migrant women in reshaping the urban environment. By ascribing significance, sense of security, and a sense of possibility to specific locations, these women contribute to the creation of a new urban topography. It functions also as a cartography of possibilities, illuminating the conditions under which relationships can flourish, identities can be cultivated, and experiences that have been marginalized can be recognized and made visible.

Taken together, the methods used in this study offer a comprehensive picture of how food practices operate as relational, symbolic, and transformative tools. The interviews highlight the daily organizational and interpersonal dynamics through which food promotes internal cohesion, operational efficiency, and community involvement. The ecological maps visually reinforce these findings by placing the restaurant within a broad network of actors, highlighting its role in the urban fabric. The photointervention adds an experiential dimension of the trainees to the analysis, revealing how participation in the project contributes, through food, to building the women’s identity, strengthening their capacity to act, and generating new forms of mutual support. Finally, the World Café and the participatory affective map extend this analysis to citizenship, showing how other food venues can also contribute to generating a sense of belonging, facilitating encounters between different people, and reconfiguring perceptions of accessibility and inclusion. A consistent pattern emerges across all methods: food simultaneously serves as a language, a resource, and a catalyst for individual and collective transformation.

## 4. Discussion and Conclusions

The present study examined the case of a social restaurant as a community tool capable of promoting well-being, recognition, empowerment, and social transformation through the food practices on which it is based, showing how these practices function as a medium for identity negotiation, cultural belonging, and the exercise of migrant agency within everyday interactions. As emerged from participants’ accounts and visual materials, these dynamics were enacted through concrete, situated food-related practices. The integrated analysis of various qualitative sources has highlighted frequently overlooked ways in which community building processes are fostered in everyday life (Corrales-Øverlid, 2023; Lugosi et al., 2023). Far from being neutral or purely instrumental activities for the satisfaction of basic needs, the findings show that food practices act as vehicles for generating relationships, belonging, and transformation, revealing a situated, embodied, and innovative way of community-building (Rivera-Navarro et al., 2020). Within this relational dynamic, food also emerges as a form of care, expressed, for example, through gestures of mutual support in the kitchen or attentive recognition of each other’s needs (flourishing with and for each other), elements that were consistently reflected in the different strands of the results. The analysis thus underscores the efficacy of this restaurant as a paradigm for the adept management of quotidian multiculturalism through the medium of food and associated practices. In this sense, everyday multiculturalism is understood as the set of interactions, negotiations, and relational gestures at the micro level that take place in the daily flow of work, learning, and coexistence. The daily practices in the restaurant exemplify how cultural differences are managed, made understandable, and transformed within ordinary situations, ranging from the adaptation of recipes to collaborative cooking, from service encounters with customers to shared moments of culinary storytelling. This emerged clearly in the narratives and observational data, where participants recounted everyday negotiations occurring during cooking, serving, and interacting with customers. These exchanges are fundamental and demonstrate how multiculturalism is put into practice, not as an abstract principle, but as a continuous form of relational governance that allows participants to recognize each other, address tensions, and co-create new shared cultural repertoires. Firstly, the practices of care through food present serve to reinstate dignity and value to the frequently unrecognized knowledge of migrant women, thereby enabling participants to redefine themselves as competent, visible in the public sphere, and professionally active (Pennisi et al., 2020; Cazorla-Becerra et al., 2025). In this sense, the community model can generate an enabling environment that allows for the coexistence and articulation of previous, new, and future identities for migrants (Iyer et al., 2009; Jetten et al., 2010). Secondly, the acquisition of social recognition and affirmation in the new country, attributable to the restaurant and food, occurs not only within the working community but also extends to the surrounding area, thereby reinforcing the bond between the participants and the local context (Malberg Dyg et al., 2020; Kudejira, 2021). It is precisely the food and menu at the restaurant that creates opportunities for intercultural contact during meals between the local community and migrant women participating in the program.

These results prompt reflections in the field of community psychology, encouraging the recognition of the importance of socio-material spaces. In accordance with Patgiri’s (2022) observations, locales that organize their undertakings around food constitute political and emotional domains wherein roles are deliberated, meaningful relationships are cultivated, and quotidian manifestations of resistance and transformation are instigated. Furthermore, it has been confirmed that catering activities involving migrants contribute significantly to the mental health and quality of life of migrants themselves in their new country (Silva & Pereira, 2023; Lillekroken et al., 2024; Schuch & Wang, 2015). As emphasized by Alfadhli and Drury (2018), collective practices have the potential to engender a sense of empowerment, provided they facilitate mutual recognition, active participation, and the capacity to influence one’s own environment. In line with Maton and Salem’s (1995) theoretical framework, the thematic and narrative analysis suggest that the social restaurant operates as a community context that promotes empowerment. It promotes growth-oriented values, offers accessible and meaningful roles, provides peer support, and is characterized by inclusive and shared leadership. The project analyzed seems to fully embody these dimensions through food and collective eating practices.

This study expands previous literature also by integrating visual and dialogic methodologies into the analysis of women’s collective action in migratory contexts, showing how these tools generate situated knowledge while strengthening community bonds and participants’ political agency. The findings suggest practical pathways for designing community interventions that recognise and value food-related practices as spaces of care and identity reconstruction. They also hold potential for informing training processes within interdisciplinary teams working with migrant who share similar trajectories. Finally, the insights produced here may contribute to local policy development aimed at reinforcing networks of solidarity-based economies and mutual support among migrant women, valuing their skills, experiences, and central role in community sustainability.

The analysis of the results confirms the relevance of food practices as tools for intercultural mediation, spaces for agency, and opportunities for re-signification of identity and food as a “platform for expressing multiple and hybrid identities and belonging, as well as for claiming social presence and visibility” (Corrales-Øverlid, 2023, p. 9). In this study, identity construction emerges through the everyday food practices that participants use to express, negotiate, and reinterpret personal and cultural meanings within the social restaurant. The practices observed highlight this transformative potential: cooking, serving, and talking about a traditional dish become acts capable of subverting stigmatising narratives about migration, valuing often invisible skills, and building relationships. In this sense, food practices also take the form of everyday micro-political acts, capable of influencing and generating more inclusive and relational forms of citizenship.

It is therefore essential that programmes and structures are put in place to support the continuity of such projects, in recognition of the value of these food places. While the under-analysis project is an exemplar of commendable community practice, a more profound and critical reflection is also necessary on its structural limitations, long-term sustainability, and genuine capacity to exert an impact at the collective level. In the absence of adequate institutional support, similar initiatives risk remaining limited experiences, dependent on the commitment of individuals, and being experienced as places of temporary inclusion rather than structures capable of producing lasting change. Moreover, the symbolic recognition gained in the workplace and community does not invariably translate into concrete future opportunities for all, such as access to stable career paths, active citizenship, or political inclusion. It is therefore essential that such practices do not remain relegated to individual or marginal community experiences, but are recognized as transformative mechanisms that deserve continuity, investment, and recognition in social and urban policy plans. Finally, from a practical standpoint, the findings offer valuable guidance for a range of community-based initiatives. For example, they may inform European contexts in which similar activities are being developed: community centres or social canteens; cooperatives focused on the production and sale of food, where work relations, socio-labour autonomy, and mutual support are reconfigured; state or NGO programmes aimed at strengthening community networks around food sovereignty, collective health, or the socio-labour integration; and educational or participatory projects that promote visual or creative methodologies to explore experiences of integration, care, and identity in intercultural settings.

## 5. Limits and Future Research

This study has limitations that are important to recognize. First, as a single case study conducted in a specific context, the situated and contextual nature precludes the possibility of generalising the results to other contexts. Although this aspect, its analytical insights can still be transferred to other community initiatives operating in the social and solidarity economy: by focusing on the processes identified and the contextual descriptions provided in the study, it is possible to identify conceptual and practical guidelines that could be effectively applied in similar contexts. Secondly, the data collection process predominantly focused on the perspectives of the operators and female trainees involved in the project. However, it did not systematically incorporate other relevant perspectives, such as those of clients or institutional actors. These voices could enhance the comprehension of the dynamics instigated by the project, facilitating a more ecological interpretation. Another potential limitation concerns potential selection bias. Participants who consented to participate in activities may have been those with particularly positive experiences of the project or with a strong sense of belonging to the restaurant. Although every effort was made to include different perspectives, it is possible that those who were less satisfied or less involved were underrepresented, limiting the range of views collected. Finally, the researcher’s proximity to the context and the participatory nature of the research, while a strength in terms of ecological relevance, also require attention. In this sense, the contribution of the others authors helped mitigate possible interpretative biases, which, however, cannot be entirely ruled out.

Future research could explore how similar projects fit into broader territorial networks, questioning how community food practices can be supported and institutionalized, what factors most influence the success and sustainability of projects, and what role community psychology can play in facilitating the transition from local practices to systemic transformative devices. In this regard, community psychology is required to extend beyond the conventional boundaries of its domains of intervention and application, embracing novel social challenges that signify pivotal sites of transformation. This suggests the necessity for enhanced communication with public institutions, local authorities, and stakeholders involved in territorial food policies, with the objective of contributing to the establishment of more equitable and inclusive food systems. Finally, it would be beneficial to investigate the educational, therapeutic, and political potential of food as a community language. Longitudinal studies could facilitate a more comprehensive exploration of the participants’ identity and professional trajectories over time, in addition to assessing the long-term impact of their involvement in the project on their lives and relationships.

## Figures and Tables

**Table 1:** Sociodemographic characteristics of the participants in the staff interviews.

ID Code	Age	Gender	Place of Birth	Years in Italy at the Time of Data Collection	Role in the Restaurant	Time in the Restaurant (Months)
01	47	Female	Chile	2011	Program Coordinator	12
02	28	Female	Albania	2019	Communication Manager and Program Assistant	24
03	27	Female	Italy	NA	Trainee	1
04	57	Female	Morocco	2004	Dining Room Assistant	26
05	28	Female	Missouri, USA	2018	Assistant Restaurant Manager	17
06	33	Female	Guinea	2014	Sous-chef	36
07	31	Female	USA	2017	President	48
08	30	Female	Ukraine	2016	Trainer Chef	18

**Table 2:** Sociodemographic data of photointervention participants at two stages of the training program (T1 = beginning; T2 = end).

Group 1 (Start of the Training)				Group 2 (End of the Training)			
Age	Gender	Place of Birth	Years in Italy at the Time of Data Collection	Age	Gender	Place of Birth	Years in Italy at the Time of Data Collection
32	Female	Pakistan	15	54	Female	Morocco	40
41	Female	Morocco	20	32	Female	Pakistan	10
43	Female	Ghana	12	32	Female	Nigeria	2
46	Female	Ghana	17	51	Female	Morocco	22
26	Female	Mexico	3	30	Female	Tunisia	9

**Table 3:** Sociodemographic characteristics of participants involved in the World Café and affective mapping activities.

ID Code	Age	Gender	Place of Birth	Years in Italy at the Time of Data Collection	Employment
01	25	Female	Italy	25	Marketing communications manager
02	28	Female	Italy	28	Teacher
03	27	Female	Italy	27	Student
04	33	Female	Peru	1	Unemployed
05	63	Female	Italy	63	Retired
06	63	Female	Italy	63	Retired
07	63	Female	Italy	63	Retired
08	54	Female	Morocco	40	Unemployed
09	45	Female	Indonesia	2	Employee
10	46	Female	Argentina	2	Retired
11	53	Female	Italy	53	Tourist reception
12	36	Female	Morocco	0.3	Waitress
13	25	Female	Guinea	1	Student
14	21	Female	Bangladesh	3	Student
15	40	Female	Bangladesh	3	Homemaker
16	65	Female	Italy	65	Council member
17	39	Female	Italy	39	Municipal employee

**Table 4:** Main themes and subthemes emerging from the semi-structured interviews conducted with staff members.

Technique	Themes	Subthemes
**Semi-structured interviews with staff**	Food as a central feature of the project: from personal experience to social interaction	- Food as a central aspect of the project. - Food as a language. - Food assumes a ritualized and formalized role.
	Food as an operational and regulative tool within the internal community of the project	- Food as a “third device”: organization of work in the kitchen, maintaining the collective goal of the project. - Food and food practices as operational strategies: informal moments of food sharing, playful aspect, dialogue, storytelling.
	From kitchen to city: exploring and transforming the larger community through food	- Openness, exploration, and change: from individual to community standpoint. - Transformative potential.
	Food as a space for agency: practices of self-determination and identity transformation	- Reaffirm the sense of identity. - Empowerment of migrant women trainees. - Cooking as a space for recognition and knowledge reconfiguration.

**Table 5:** Main themes and subthemes emerging from the ecological maps conducted with staff members.

Technique	Themes	Subthemes
**Ecological Maps**	Relational positioning in the local community	- Key institutional partners - Supportive networks - Critical/fragile connections

**Table 6:** Main themes and subthemes emerging from the Photo Intervention, organized across both participant groups.

Technique	Group	Themes	Subthemes
**Photo-intervention**	T1	Personal development and future opportunities	- Satisfaction, happiness, and self-efficacy - Humility and patience, effort and consistent learning
	T2	A fresh start through self-growth	- Food practices as transformation and rebirth - Empowering experience for independence, opportunities, and social inclusion
	T1	Food as a relational and cohesive space	- Women’s empowerment through connection and collaboration
	T2	Flourishing with and for each other	- Teamwork and a sense of family - Making compromises and working hard to provide stability - Gift, protection, and womens’ mutual support
	T1	Harmony and richness overcoming differences	- A core and unified root in diversity - Understanding different priorities
	T2	Food as a way of nourishing diversity and promoting dialogue	- Food as a love language beyond cultural and personality barriers - Creating new shared roots through communication and unity - Emphasizing differences through creativity and self-expression
	T1	Bringing one’s origins on the table	- Discovery and exploration through tasting and ingredients - Feeling at home in Italy - Visibility within the local community
	T2	Connections with the local community	- Promoting integration by mixing old and new flavours - Representation and responsibility

**Table 7:** Main discussion themes emerging from the World Café, subsequently represented visually on the map.

Technique	Discussion Themes	Subthemes
**World café and participatory affective cartography**	Characteristics of nurturing places	- Relational and symbolic welcoming - Recognition and legitimacy in the urban context - Barriers to accessing supportive urban spaces
	Urban spaces enabling (or limiting) migrant reception and inclusion	- Supermarkets as linguistically safe spaces - Markets as spaces for informal intercultural exchange - Fragmented opportunities for socialization
	Food places as spaces for community building	- Family-evocative food spaces - Women-run food places as points of representation - Institutional spaces promoting Intercultural socialization

## Data Availability

The dataset used is currently not openly available in order to guarantee the privacy of the participants involved in the study and not to violate public dissemination of information that could identify participants and violate privacy regulations and ethical standards regarding the protection of personal data.
